# Associations of Health Belief Model Constructs and Self-Control with Drug Avoidance Intention Among University Students in the Republic of Korea: A Cross-Sectional Study

**DOI:** 10.3390/bs16091653

**Published:** 2026-09-15

**Authors:** Jungmin Lee, Su Jung Lee

**Affiliations:** School of Nursing, Research Institute of Nursing Science, Hallym University, Chuncheon 24252, Gangwon-do, Republic of Korea; j_lee0624@hallym.ac.kr

**Keywords:** students, health belief model, self-control, intention, primary prevention

## Abstract

Drug use among young adults in the Republic of Korea has increased sharply, yet prevention at the university level remains limited, and the determinants of drug avoidance intention are poorly understood. Guided by the Health Belief Model extended with self-control, this study examined the associations of five health belief constructs and self-control with drug avoidance intention. A cross-sectional online survey was conducted among 280 university students aged 19–26 years. Drug avoidance intention, health beliefs, and self-control were assessed using validated instruments. Hierarchical multiple linear regression was used to test the hypothesized associations, and exploratory indirect-effect analyses were conducted for each Health Belief Model construct using 5000 bootstrap resamples, with robustness models adjusting for the remaining constructs. The mean drug avoidance intention score was 4.22, and the final model explained 58.5% of the variance. Drug refusal self-efficacy, perceived severity, perceived benefits, and self-control were positively associated with drug avoidance intention, whereas perceived barriers were negatively associated; perceived susceptibility, for which no directional hypothesis was specified, was not significant. Exploratory robustness analyses indicated indirect associations through self-control for perceived susceptibility and perceived benefits after adjustment for the remaining Health Belief Model constructs. These findings indicate that drug avoidance intention was associated with both health beliefs and self-regulatory capacity. Longitudinal and intervention studies are needed.

## 1. Introduction

Illicit drug use and non-medical use of prescription medications are global public health concerns associated with substantial health and social burdens, including substance use disorders and overdoses. The United Nations Office on Drugs and Crime estimated that approximately 316 million individuals aged 15–64 years worldwide used drugs other than alcohol and tobacco in 2023 (United Nations Office on Drugs and Crime, 2025). Although trends have not been uniform across all substances, the use of cannabis and hallucinogens among university students and other emerging adults increased or remained at high levels, along with the spread of new modes of use (Patrick et al., 2024). University students are generally in emerging adulthood, a period marked by greater autonomy in decision-making and increasing independence (Arnett, 2016). The transition to university may involve changes in living environments and social relationships (Worsley et al., 2021). Recent studies among university students documented the use of multiple illicit substances and the associated harm (Boden & Day, 2023); however, prevalence estimates vary across studies (Aslan & Önal, 2024).

The Republic of Korea provides a timely context for prevention research among university students. A joint announcement by the Ministry of Education and the Ministry of Food and Drug Safety reported that the number of drug offenders in their twenties increased from 3521 in 2019 to 8368 in 2023, a 138% increase, and that this age group accounted for the largest proportion of identified drug offenders in 2023 (30%) (Ministry of Education & Ministry of Food and Drug Safety, 2024). In response, the government announced plans to expand university participation in drug-prevention activities and to develop prevention-education guidelines for university students (Ministry of Education & Ministry of Food and Drug Safety, 2024). These initiatives indicate that drug prevention among young adults has become an emerging policy priority, while university-level prevention remains in a phase of further development rather than being uniformly institutionalized. In addition, a nationwide Korean survey documented substantial stigma and discriminatory attitudes toward people with drug addiction (Jang et al., 2023). Together, these epidemiological, educational, and social conditions make the Korean university setting relevant for examining how students form intentions to avoid drug use.

Research on drug use among university students has largely followed two lines. One documents the prevalence, correlates, and harms of illicit drug use and the non-medical use of prescription cognitive enhancers (Boden & Day, 2023; Sharif et al., 2021). The other evaluates campus-based prevention interventions, for which some beneficial outcomes have been reported, although the evidence remains limited and heterogeneous in intervention content and outcome measures (Dick et al., 2019; Ghani et al., 2024). These bodies of work are important for characterizing drug use and evaluating existing programs, but they provide less information about the modifiable psychological factors associated with preventive intention before drug use occurs. Drug avoidance intention—the desire, commitment, and readiness to refrain from drug use (Suwanchinda et al., 2019)—is relevant in this context because it can be assessed among students regardless of prior use and represents a proximal psychological antecedent of behavior, although intention does not ensure subsequent behavior (Webb & Sheeran, 2006). Identifying the factors associated with drug avoidance intention may therefore help clarify which psychological constructs warrant evaluation in future prevention studies.

The Health Belief Model (HBM) provides a framework for examining such factors through perceived susceptibility, perceived severity, perceived benefits, perceived barriers, and self-efficacy (Taflinger & Sattler, 2024). HBM-based education among adolescents has been associated with changes in drug-prevention beliefs and preventive behaviors (Fadaei et al., 2020). A web-based intervention among young adults reported improvements in prevention self-efficacy and drug avoidance intention (Chang & Chen, 2023). Drug refusal self-efficacy has been associated with drug avoidance intention among adolescents (Latt et al., 2024). Among Korean adults, HBM-related beliefs have also been associated with self-assessed likelihood of future drug use (Yang et al., 2025). Importantly, the constructs are conceptually distinct: perceived severity concerns the seriousness of drug-related consequences, perceived benefits and barriers concern evaluations of avoidance or prevention, and drug refusal self-efficacy concerns confidence in refusing drugs when they are offered. Accordingly, examining the HBM constructs separately can identify whether different beliefs show different associations with drug avoidance intention rather than assuming that health beliefs operate as a single undifferentiated factor.

HBM constructs primarily represent health-related appraisals and behavior-specific beliefs (Taflinger & Sattler, 2024). They do not explicitly capture a broader self-regulatory capacity to inhibit impulses and maintain goal-directed behavior across situations. Self-control reflects this general capacity to regulate impulses and immediate temptations in accordance with longer-term goals (Tangney et al., 2004). This distinction is important because drug refusal self-efficacy and self-control are related but not equivalent: the former reflects confidence in performing a specific refusal behavior, whereas the latter reflects a more general capacity for self-regulation. Self-control has been examined as a self-regulatory characteristic relevant to illicit substance use and related harm in young adulthood (Greenwood et al., 2025). Low self-control has also been examined prospectively in relation to e-cigarette use intentions and subsequent use among adolescents (Sutherland et al., 2022). Among undergraduate students, e-cigarette use has been examined using a dual-process framework that incorporates both reflective and non-conscious processes (Phipps et al., 2024). Thus, adding self-control to the HBM framework addresses an explanatory question that health beliefs alone cannot fully answer: whether general self-regulatory capacity is associated with drug avoidance intention beyond students’ specific beliefs about drug-related risk and avoidance.

This distinction may be particularly relevant in the Korean university context. Recent research among Korean university students reported low self-reported drug use but found that self-control and social stigma were associated with drug-permissive attitudes (Lee et al., 2024), suggesting that prevention-related cognition and self-regulation may both be relevant in this setting. More broadly, the coexistence of increasing concern about drug use among young adults, developing university-level prevention initiatives, and substantial social stigma may shape how Korean students evaluate drug-related risk and avoidance. Examining HBM constructs together with self-control therefore allows the study to assess two conceptually distinct domains—health-related beliefs and general self-regulatory capacity—within this specific prevention context.

**Figure 1:**
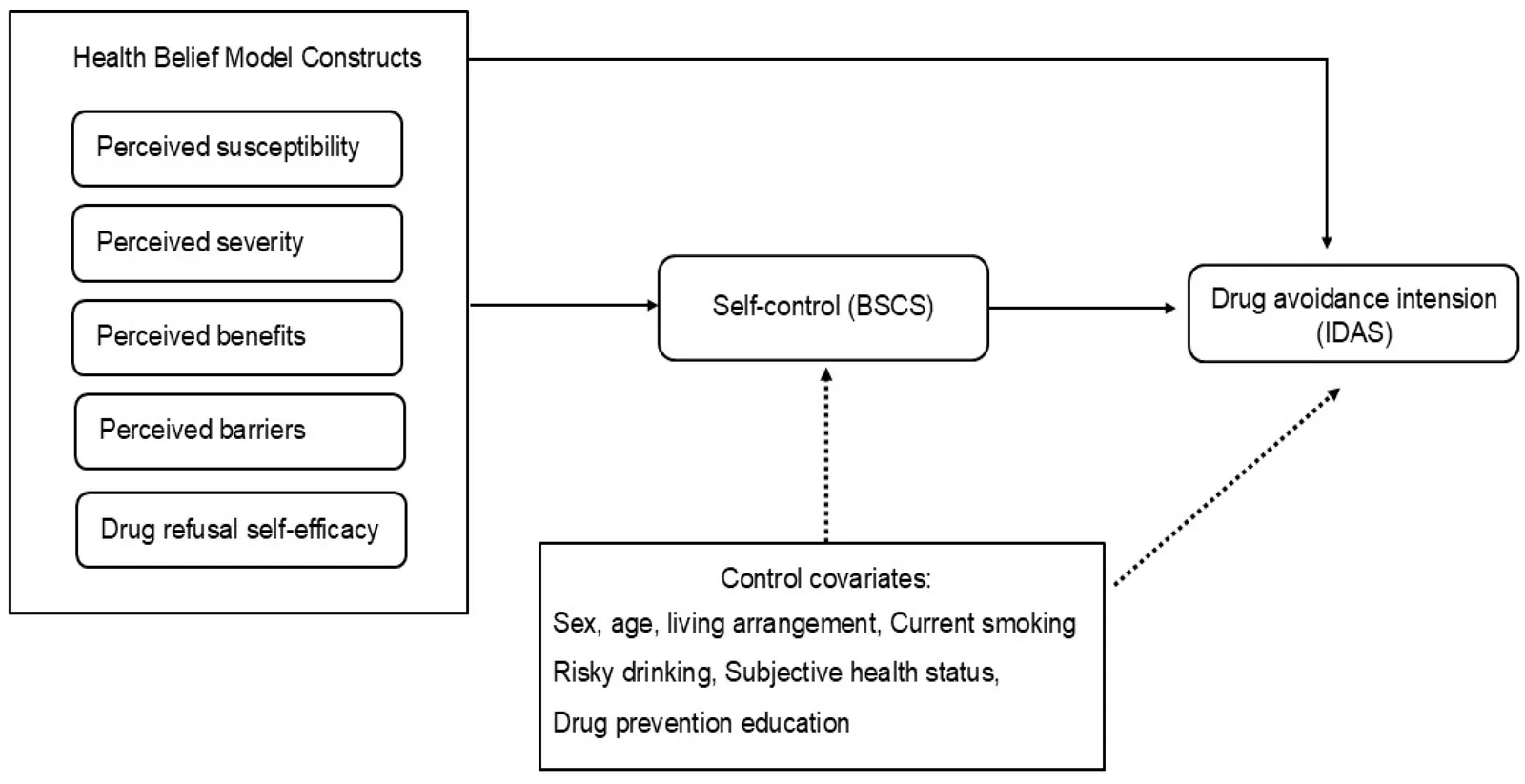
Conceptual framework and analytic model of the study. Abbreviations: BSCS, Brief Self-Control Scale; HBM, Health Belief Model; IDAS, Intention to Drug Avoidance Scale. Solid arrows represent the hypothesized directional associations for perceived severity, perceived benefits, perceived barriers, drug refusal self-efficacy, and self-control; perceived susceptibility was examined without a directional hypothesis. The pathway through self-control represents the exploratory indirect-effect analyses. Dotted arrows do not represent hypothesized associations; they indicate the covariate adjustment applied in the analyses. All regression and exploratory indirect-effect models were adjusted for sex, age, living arrangement, current smoking, risky drinking, subjective health status, and drug prevention education.

Accordingly, this study examined the associations of perceived susceptibility, perceived severity, perceived benefits, perceived barriers, drug refusal self-efficacy, and self-control with drug avoidance intention among university students in the Republic of Korea. We hypothesized that perceived severity (H1) and perceived benefits (H2) would be positively associated with drug avoidance intention, whereas perceived barriers (H3) would be negatively associated. Drug refusal self-efficacy (H4) was hypothesized to be positively associated with drug avoidance intention. We further hypothesized that self-control would be positively associated with drug avoidance intention after adjustment for all five HBM constructs (H5). Perceived susceptibility was examined without a directional hypothesis because, in the instrument used in this study, its items assess respondents’ perceived likelihood of encountering or becoming involved with drugs rather than susceptibility to harm conditional on drug use; previous Korean evidence has also linked higher perceived susceptibility to a higher self-assessed likelihood of future drug use (Yang et al., 2025). Finally, because the data were cross-sectional and the temporal ordering of the variables could not be established, indirect associations between each HBM construct and drug avoidance intention through self-control were examined exploratorily rather than as tests of causal mediation. The conceptual framework and analytic model are presented in Figure 1.

## 2. Materials and Methods

### 2.1. Study Design

This study used a cross-sectional web-based survey design to examine the associations of Health Belief Model constructs and self-control with drug avoidance intention among undergraduate students in the Republic of Korea. Reporting of the web-based survey procedures was guided by the Checklist for Reporting Results of Internet E-Surveys (CHERRIES) (Eysenbach, 2004), and a completed checklist is provided in Supplementary Table S3.

### 2.2. Participants and Recruitment

The target population comprised undergraduate students aged 19–26 years who were currently enrolled in universities in the Republic of Korea. Participants were recruited through Macromill Embrain (Seoul, Republic of Korea), a commercial non-probability, opt-in online research panel. The provider maintains a nationwide panel of approximately 1.8 million registered members, with panel members distributed across all major regions of the Republic of Korea. Targeted survey invitations were sent to registered panel members aged 19–26 years based on age information in the panel database.

Eligibility criteria were age 19–26 years, current enrollment as an undergraduate student, and the ability to complete an online questionnaire in Korean. At survey entry, university-student status was verified using a screening question on occupation. Respondents who did not select “university student” were automatically screened out and could not proceed to the main questionnaire. Initial data collection was conducted from 21 to 27 April 2026. Following approval of an Institutional Review Board protocol amendment to address the sex imbalance observed during the initial recruitment, 60 additional female participants were recruited from 16 to 17 July 2026. The final analytic sample comprised 280 participants. Participants who completed the survey received panel reward points in accordance with the provider’s standard compensation policy based on survey completion time.

### 2.3. Web Survey Administration and Data Quality

The survey was administered as a closed web survey to invited panel members. Before formal data collection, the questionnaire was pre-tested by the research team to confirm its functionality and survey flow. The questionnaire comprised 51 screens; responses to all items on a screen were required before participants could proceed, although participants could return to previous screens to modify their responses or discontinue the survey at any time. Duplicate participation was restricted through a unique panel identification system. The panel provider also applied predefined data-quality screening procedures, including checks for repetitive identical responses and inattentive or implausible responses. Detailed web-survey administration and data-quality procedures are reported in the completed CHERRIES checklist (Supplementary Table S3).

### 2.4. Ethical Considerations

The study protocol and subsequent amendment were reviewed and approved by the Institutional Review Board of Hallym University (HIRB-2025-136 and HIRB-2025-136-R, respectively). Before beginning the survey, all potential participants received online information explaining the study’s purpose, procedures, the voluntary nature of participation, confidentiality, and the right to discontinue participation without penalty. Electronic informed consent was obtained before access to the questionnaire was permitted, and only individuals who provided consent were allowed to participate. The analytic dataset available to the investigators contained no personally identifiable information. Contact information for professional addiction counseling services was provided in case participants experienced discomfort related to the survey content. All study data were stored on password-protected electronic devices.

### 2.5. Measures

#### 2.5.1. Drug Avoidance Intention

Drug avoidance intention was measured using the Korean version of the 22-item Intention to Drug Avoidance Scale (IDAS), originally developed and psychometrically evaluated for Thai adolescents (Suwanchinda et al., 2019). This scale assesses desire, commitment, and readiness to avoid drug use. Each item is rated on a 5-point Likert scale, with higher mean scores indicating a stronger drug avoidance intention. The original scale demonstrated high internal consistency, with a Cronbach’s α of 0.94. The Korean version was translated, culturally adapted, and psychometrically evaluated in a separate study. In this study, Cronbach’s α was 0.954.

#### 2.5.2. Health Beliefs Regarding Drug Use

Health beliefs regarding drug use were assessed using the 15-item instrument developed by Yang et al., based on the Health Belief Model, with permission from the developers (Yang et al., 2023). The instrument comprises five subscales, with three items each: perceived susceptibility, perceived severity, perceived barriers, perceived benefits, and drug refusal self-efficacy. Items were rated on a 5-point Likert scale ranging from 1 (strongly disagree) to 5 (strongly agree), with higher mean scores indicating higher levels of the corresponding health belief construct. The perceived susceptibility items assessed respondents’ appraisal of their own likelihood of encountering or becoming involved with drugs, rather than their vulnerability to harm conditional on use.

In the original study, Cronbach’s α values were 0.860 for perceived susceptibility, 0.915 for perceived severity, 0.672 for perceived barriers, 0.848 for perceived benefits, and 0.838 for self-efficacy. In this study, Cronbach’s α values were 0.821, 0.901, 0.628, 0.752, and 0.713, respectively.

#### 2.5.3. Self-Control

Self-control was assessed using the Korean version of the 13-item Brief Self-Control Scale (BSCS), originally developed by Tangney et al. (2004) and validated in Korean by Hong et al. (2012). This scale assesses an individual’s capacity to regulate impulses, resist temptation, and maintain goal-directed behavior. Items are rated on a 5-point Likert scale ranging from 1 (strongly disagree) to 5 (strongly agree). Nine negatively worded items were reverse-coded, with higher mean scores indicating greater self-control. Cronbach’s α in this study was 0.877.

#### 2.5.4. Sociodemographic and Behavioral Characteristics

Sociodemographic and behavioral characteristics included sex, age, living arrangements, subjective health status, perceived stress level, current smoking, risky drinking, and drug prevention education. Living arrangements were assessed as living with parents, alone, or in a dormitory. Regarding group comparisons and regression analyses, living alone and in dormitory residences were combined into an independent-living category. Subjective health status was classified as poor, fair, or good, and perceived stress was classified as low, moderate, or high. Current smoking status and drug prevention education were assessed as yes or no, respectively. Risky drinking was defined as consuming seven or more drinks for men or five or more drinks for women on a single occasion at least twice per week (Kim et al., 2026).

### 2.6. Statistical Analysis

Statistical analyses were performed using IBM SPSS Statistics (version 29.0; IBM Corp., Armonk, NY, USA). Statistical significance was set at a two-tailed *p* < 0.05. Frequencies, percentages, means, and standard deviations were calculated to summarize participant characteristics and the main study variables. Differences in drug avoidance intention according to participant characteristics were examined using Welch’s *t*-tests and one-way analysis of variance, followed by Bonferroni post hoc comparisons when appropriate. Pearson correlation coefficients were calculated to examine correlations among drug avoidance intention, the five Health Belief Model constructs, and self-control.

Hierarchical multiple linear regression was conducted to examine factors associated with drug avoidance intention. Sex, age, living arrangement, current smoking, risky drinking, subjective health status, and drug prevention education were entered into Model 1. The five Health Belief Model constructs—perceived susceptibility, perceived severity, perceived barriers, perceived benefits, and drug refusal self-efficacy—were entered into Model 2, and self-control was added in Model 3. Living alone and dormitory residence were combined as independent living. Subjective health status was entered as a categorical variable with good health as the reference category. Multicollinearity was assessed using variance inflation factors, and independence of residuals was evaluated using the Durbin–Watson statistic.

Exploratory indirect-effect analyses were conducted using PROCESS for SPSS (Model 4), with self-control specified as the intervening variable between each Health Belief Model construct and drug avoidance intention (Hayes, 2022). In the initial construct-specific models, each Health Belief Model construct was entered separately as the independent variable. Because these models do not isolate construct-specific indirect associations independently of the remaining health beliefs, robustness models were additionally estimated in which the other four Health Belief Model constructs were included as covariates in both the self-control and outcome equations. All indirect-effect models were adjusted for sex, age, living arrangement, current smoking, risky drinking, subjective health status, and drug prevention education. Indirect effects were estimated using 5000 bootstrap resamples with 95% confidence intervals (CIs) and were considered statistically significant when the bootstrap CI did not include zero. Results of the robustness models are presented in Supplementary Table S2.

## 3. Results

### 3.1. Participant Characteristics and Descriptive Statistics

In total, 280 undergraduate students were included in the analysis. Their mean age was 21.7 years (SD = 1.9), and 170 participants (60.7%) were male. Most participants lived with their parents (*n* = 174, 62.1%), and 70 (25.0%) had received drug prevention education. The mean drug avoidance intention score was 4.22 (SD = 0.61). Additional descriptive statistics are presented in Table 1.

### 3.2. Differences in Drug Avoidance Intention by Participant Characteristics

Drug avoidance intention differed significantly according to sex, subjective health status, current smoking status, and risky drinking habits (Supplementary Table S1). Male participants had higher drug avoidance intention than female participants (4.30 vs. 4.11, *p* = 0.020). Nonsmokers had higher scores than current smokers (4.26 vs. 3.97, *p* = 0.015), and participants without risky drinking habits had higher scores than those with risky drinking habits (4.24 vs. 3.74, *p* = 0.041). Additionally, drug avoidance intention differed according to subjective health status (*p* = 0.026), with the fair health group exhibiting a lower score than the good health group in the post hoc comparison. No statistically significant differences were observed in living arrangements, perceived stress, or drug prevention education.

### 3.3. Correlations Among the Main Study Variables

Drug avoidance intention was positively correlated with drug refusal self-efficacy (r = 0.67, *p* < 0.001), perceived severity (r = 0.53, *p* < 0.001), perceived benefits (r = 0.39, *p* < 0.001), and self-control (r = 0.33, *p* < 0.001) and negatively correlated with perceived susceptibility (r = −0.50, *p* < 0.001). Perceived barriers were not significantly correlated with drug avoidance intention (r = −0.05, *p* > 0.05; Table 2).

### 3.4. Factors Associated with Drug Avoidance Intention

Table 3 presents the hierarchical multiple regression analysis results. Sociodemographic and behavioral characteristics explained 9.8% of the variance in drug avoidance intention in Model 1. The addition of the five Health Belief Model components in Model 2 increased the explained variance by 47.8% (ΔR^2^ = 0.478, F change = 59.91, *p* < 0.001). Adding self-control in Model 3 resulted in an increase of 0.9% (ΔR^2^ = 0.009, F change = 6.01, *p* = 0.015).

The final model explained 58.5% of the variance in drug avoidance intention (adjusted R^2^ = 0.563; F(14, 265) = 26.69, *p* < 0.001). Drug refusal self-efficacy (B = 0.410, 95% CI [0.302, 0.519], *p* < 0.001), perceived severity (B = 0.220, 95% CI [0.129, 0.310], *p* < 0.001), perceived benefits (B = 0.117, 95% CI [0.032, 0.202], *p* = 0.007), and self-control (B = 0.108, 95% CI [0.021, 0.196], *p* = 0.015) were positively associated with the intention to avoid drug use. Perceived barriers were negatively associated with drug avoidance intention (B = −0.097, 95% CI [−0.164, −0.029], *p* = 0.005). Independent living was positively associated with drug avoidance intention compared to living with parents (B = 0.144, 95% CI [0.042, 0.246], *p* = 0.006). Perceived susceptibility was not statistically significant in the final model (B = −0.077, 95% CI [−0.155, 0.001], *p* = 0.053).

### 3.5. Exploratory Indirect-Effect Analyses

Table 4 presents the exploratory indirect effect analysis results. Significant indirect effects through self-control were observed for perceived susceptibility (indirect effect = −0.052, 95% bootstrap CI [−0.085, −0.023]), perceived severity (indirect effect = 0.042, 95% bootstrap CI [0.015, 0.076]), perceived benefits (indirect effect = 0.062, 95% bootstrap CI [0.030, 0.101]), and drug refusal self-efficacy (indirect effect = 0.045, 95% bootstrap CI [0.018, 0.073]). Perceived barriers’ indirect effect was not statistically significant (indirect effect = −0.014, 95% bootstrap CI [−0.052, 0.018]). In robustness models adjusting for the remaining four Health Belief Model constructs, the indirect effects through self-control remained statistically significant for perceived susceptibility (indirect effect = −0.023, 95% bootstrap CI [−0.055, −0.003]) and perceived benefits (indirect effect = 0.023, 95% bootstrap CI [0.003, 0.048]). The indirect effects for perceived severity and drug refusal self-efficacy were no longer statistically significant, whereas the indirect effect for perceived barriers remained non-significant (Supplementary Table S2).

## 4. Discussion

This study examined the associations of Health Belief Model constructs and self-control with drug avoidance intention among university students in the Republic of Korea. As hypothesized, drug refusal self-efficacy, perceived severity, perceived benefits, and self-control were associated with higher drug avoidance intention, whereas perceived barriers were associated with lower drug avoidance intention. Perceived susceptibility was not significantly associated with drug avoidance intention after considering the other Health Belief Model constructs and self-control. In the exploratory indirect-effect analyses, several construct-specific indirect associations through self-control were observed; however, after adjustment for the remaining Health Belief Model constructs, only those for perceived susceptibility and perceived benefits remained statistically significant. Overall, these findings suggest that drug avoidance intention was associated with both health-related beliefs and self-regulatory characteristics, while the individual Health Belief Model constructs showed distinct patterns of association. The inclusion of the Health Belief Model constructs substantially improved the explanatory capacity of the model. This finding suggested that cognitive evaluations of drug-related risks and avoidance behaviors were closely associated with drug avoidance intention, even after considering sociodemographic and behavioral characteristics. The Health Belief Model distinguishes between perceived susceptibility, severity, benefits, barriers, and self-efficacy in explaining health-related decision-making (Alyafei & Easton-Carr, 2024). Among drug-related studies, health beliefs have been associated with self-assessed likelihood of future drug use (Yang et al., 2025), and drug refusal self-efficacy has been associated with drug avoidance intention (Latt et al., 2024). The different patterns observed across the constructs in the present study therefore support examining them separately rather than treating health beliefs as a single overall score.

Drug refusal self-efficacy was positively associated with drug avoidance intention and showed the largest positive regression coefficient among the Health Belief Model constructs in the final model. Drug refusal self-efficacy refers to confidence in one’s ability to refuse a substance in specific situations (Wanigasooriya et al., 2021). Drug refusal self-efficacy has previously been associated with drug avoidance intention among adolescents (Latt et al., 2024). Among young adults, refusal self-efficacy has also been examined as part of prospective pathways linking affect, peer influences, and non-alcohol drug outcomes (Cheesman & Read, 2023). In the present study, drug refusal self-efficacy remained strongly associated with avoidance intention after adjustment for the other Health Belief Model constructs and self-control, whereas its adjusted indirect association through self-control was not statistically significant. This pattern is consistent with treating drug refusal self-efficacy and self-control as related but conceptually distinct constructs: the former concerns confidence in performing a specific refusal behavior, whereas the latter reflects a broader capacity for self-regulation.

Perceived severity and perceived benefits were also positively associated with drug avoidance intention. Perceived severity refers to the extent to which drug-related consequences are regarded as serious, whereas perceived benefits reflect beliefs about the value of avoiding drug use (Rosenstock et al., 1988). An HBM-based educational intervention among adolescents reported changes in drug-prevention beliefs and preventive behaviors (Fadaei et al., 2020). A web-based intervention among young adults also reported improvements in prevention self-efficacy and drug avoidance intention (Chang & Chen, 2023). The present findings therefore support the relevance of perceived severity and perceived benefits as cognitive correlates of drug avoidance intention. However, after the remaining Health Belief Model constructs were controlled, the indirect association through self-control remained significant for perceived benefits but not for perceived severity. This difference further indicates that the individual health-belief constructs should not be assumed to relate to self-control and avoidance intention in the same manner.

Perceived barriers were negatively associated with drug avoidance intention. The items used in this study addressed anticipated stigmatization during professional counseling, insufficient drug-prevention education, and low societal awareness of drug prevention (Yang et al., 2023). Barriers to substance use disorder treatment have been identified at individual, social, and structural levels (Farhoudian et al., 2022). A nationwide survey in the Republic of Korea also documented substantial negative perceptions and discriminatory attitudes toward people with drug addiction (Jang et al., 2023). These findings provide relevant context for interpreting the social and informational content of the perceived-barriers items used in the present study. However, because our study did not directly assess stigma, help-seeking behavior, or access to services, the mechanism underlying the negative association between perceived barriers and drug avoidance intention cannot be determined.

Perceived susceptibility showed a different pattern from the other Health Belief Model constructs. It was negatively correlated with drug avoidance intention at the bivariate level but was not statistically significant in the fully adjusted regression model. This finding should be interpreted in light of the way perceived susceptibility was operationalized in the instrument used in this study. The items assess respondents’ perceived likelihood of encountering or becoming involved with drugs rather than susceptibility to harm conditional on drug use (Yang et al., 2023). A study among Korean adults similarly found that higher perceived susceptibility was associated with a higher self-assessed likelihood of future drug use (Yang et al., 2025). Thus, the negative bivariate association may partly reflect perceived proximity to drug exposure rather than the conventional HBM interpretation of vulnerability to adverse consequences. The adjusted indirect association through self-control remained statistically significant for perceived susceptibility, but this cross-sectional finding should be regarded as exploratory and should not be interpreted as evidence of a temporal or causal pathway.

Although self-control contributed to only modest additional explained variance, it remained positively associated with drug avoidance intention after all five Health Belief Model constructs were considered. Lower self-control has been associated with alcohol-related problems among college students (Lucke et al., 2024). Lower self-control has also been associated with recent cannabis use and greater frequency of cannabis use among college students (Giaquinto et al., 2024). In Korean university students, self-control has been associated with drug-permissive attitudes (Lee et al., 2024). Impulsivity and sensation seeking have also been examined in relation to drug-use intentions among young adults in the Republic of Korea (Sohn, 2025). Together, these findings support considering general self-regulatory characteristics alongside drug-specific health beliefs rather than treating the two domains as interchangeable.

The robustness analyses provide important context for the exploratory indirect-effect findings. In the initial construct-specific models, indirect associations through self-control were observed for perceived susceptibility, perceived severity, perceived benefits, and drug refusal self-efficacy, but not for perceived barriers. After adjustment for the remaining Health Belief Model constructs, only the indirect associations for perceived susceptibility and perceived benefits remained statistically significant, whereas those for perceived severity and drug refusal self-efficacy were no longer significant. This change suggests that some of the indirect associations observed in the initial models were sensitive to variance shared among the Health Belief Model constructs. Previous research has similarly examined indirect associations between self-regulatory characteristics, substance-use motives, and substance-related consequences among college students (Montgomery et al., 2024). Accordingly, the present indirect-effect findings should be interpreted as exploratory statistical associations rather than evidence that self-control causally mediates the effects of individual health beliefs.

The findings may also inform future drug-prevention research in Korean university settings. In the present sample, only 25.0% of participants reported having received drug-prevention education, and receipt of such education was not independently associated with drug avoidance intention. This finding should not be interpreted as evidence that existing university prevention education is ineffective because the variable assessed only whether education had been received and did not capture its content, duration, intensity, or quality. Rather, future evaluations of university-based prevention programs could examine whether specific constructs associated with avoidance intention—particularly drug refusal self-efficacy, perceived severity, perceived benefits, and perceived barriers—change following intervention. Given the independent association of self-control with drug avoidance intention, self-regulatory skills may also warrant evaluation as a complementary component rather than as a substitute for belief-focused prevention approaches.

A theory-based educational program has reported improvements in self-regulation and alcohol-related outcomes among adolescents (Rahimi et al., 2025). Technology-based substance-use interventions among emerging adults and college students have shown small overall beneficial effects (Hai et al., 2024). Evidence for cannabis-focused interventions among college students remains heterogeneous, with effective and ineffective approaches reported across trials (Hone et al., 2024). A systematic review and meta-analysis of digital interventions for young people likewise concluded that overall effects on substance use were generally weak, although some benefit was observed for alcohol outcomes (O’Logbon et al., 2024). Future intervention studies should therefore evaluate whether changes in specific health beliefs and self-regulatory characteristics are accompanied by changes in drug avoidance intention and, ultimately, subsequent drug-use behavior.

This study had several limitations. First, the cross-sectional design did not allow the temporal ordering of health beliefs, self-control, and drug avoidance intention to be established. The exploratory indirect-effect analyses represent statistical decompositions of concurrently measured associations and do not establish causal mediation. Although robustness models adjusted for the remaining Health Belief Model constructs, this additional adjustment does not resolve the temporal limitations of the cross-sectional design. Longitudinal studies are needed to examine whether changes in health beliefs and self-control precede changes in drug avoidance intention and subsequent behavior. Second, all variables were self-reported and may therefore have been affected by social desirability bias, recall error, and common method bias. Future studies should incorporate behavioral measures of drug refusal and prospective measures of drug-use behavior where feasible. Third, the internal consistency of the perceived barriers subscale was relatively low, and the study did not assess potentially relevant contextual factors such as peer drug use or offers, drug accessibility, actual drug exposure, or cues to action. Future studies should incorporate these factors to provide a more comprehensive account of preventive intention. Finally, participants were recruited from a non-probability, opt-in commercial online panel. The sample was not designed to represent the national distribution of university students by institution type or region, and selection into an incentivized online panel may be associated with characteristics not measured in this study. Consequently, the descriptive estimates should not be interpreted as population estimates, and the observed associations require replication using probability-based or multi-campus samples. Comparative studies across settings with different drug-use patterns, prevention systems, and social attitudes would also help determine the extent to which the observed associations are context-dependent.

## 5. Conclusions

This study found that drug avoidance intention among university students in the Republic of Korea was associated with specific health beliefs and self-control. Perceived severity, perceived benefits, and drug refusal self-efficacy were positively associated with drug avoidance intention, whereas perceived barriers were negatively associated. Self-control remained positively associated with drug avoidance intention after adjustment for all five Health Belief Model constructs. In exploratory robustness analyses, indirect associations through self-control remained significant only for perceived susceptibility and perceived benefits after adjustment for the remaining Health Belief Model constructs. These findings should be interpreted as exploratory and not as evidence of causal mediation.

Future longitudinal and intervention studies are needed to determine whether changes in these health beliefs and self-regulatory characteristics are associated with subsequent changes in drug avoidance intention and actual drug-use behavior.

## Figures and Tables

**Table 1:** Participant characteristics and descriptive statistics of major study variables (*n* = 280).

Variable	*n* (%) or Mean ± SD
Sex	
Male	170 (60.7)
Female	110 (39.3)
Age (years)	21.7 ± 1.9
Living arrangement	
With parents	174 (62.1)
Living alone	76 (27.1)
Dormitory	30 (10.7)
Subjective health	
Poor	38 (13.6)
Fair	159 (56.8)
Good	83 (29.6)
Perceived stress level	
Low	75 (26.8)
Moderate	91 (32.5)
High	114 (40.7)
Current smoking	
Yes	36 (12.9)
No	244 (87.1)
Risky drinking	
Yes	10 (3.6)
No	270 (96.4)
Drug prevention education	
Yes	70 (25.0)
No	210 (75.0)
Drug avoidance intention (IDAS)	4.22 ± 0.61
Perceived susceptibility	1.79 ± 0.85
Perceived severity	4.48 ± 0.65
Perceived benefits	4.04 ± 0.65
Perceived barriers	3.55 ± 0.77
Drug refusal self-efficacy	4.36 ± 0.63
Self-control (BSCS)	3.20 ± 0.65

Abbreviations: IDAS, Intention to Drug Avoidance Scale, BSCS, Brief Self-Control Scale.

**Table 2:** Pearson’s correlations among the major study variables (*n* = 280).

Variable	1	2	3	4	5	6	7
1. IDAS	—						
2. Susceptibility	−0.50 ***	—					
3. Severity	0.53 ***	−0.27 ***	—				
4. Benefits	0.39 ***	−0.13 *	0.34 ***	—			
5. Barriers	−0.05	0.14 *	0.17 **	0.11	—		
6. Drug refusal self-efficacy	0.67 ***	−0.56 ***	0.51 ***	0.35 ***	0.08	—	
7. BSCS	0.33 ***	−0.34 ***	0.15 *	0.29 ***	−0.12	0.29 ***	—

Abbreviations: IDAS, Intention to Drug Avoidance Scale; BSCS, Brief Self-Control Scale. * *p* < 0.05, ** *p* < 0.01, *** *p* < 0.001.

**Table 3:** Hierarchical multiple regression predicting drug avoidance intention (*n* = 280).

Panel A. Model Summary						
Model	Variables Entered	R^2^	Adj. R^2^	ΔR^2^	F-Change	*p*
Model 1	Covariates	0.098	0.071	0.098	3.67	<0.001
Model 2	Model 1+Health Belief Model components	0.576	0.555	0.478	59.91	<0.001
Model 3	Model 1+Model 2+Self-control (BSCS)	0.585	0.563	0.009	6.01	0.015
Panel B. Final model coefficients (Model 3)						
Predictor		B	SE	95% CI		*p*
Male Sex		0.064	0.052	[−0.038, 0.166]		0.219
Age		−0.010	0.013	[−0.036, 0.016]		0.434
Independent living		0.144	0.052	[0.042, 0.246]		0.006
Current smoker		−0.096	0.081	[−0.256, 0.063]		0.236
Risky drinking		−0.181	0.139	[−0.454, 0.092]		0.194
Poor subjective health		0.091	0.085	[−0.076, 0.259]		0.284
Fair subjective health		−0.006	0.059	[−0.122, 0.110]		0.923
No drug prevention education		0.010	0.060	[−0.107, 0.128]		0.862
Perceived susceptibility		−0.077	0.040	[−0.155, 0.001]		0.053
Perceived severity		0.220	0.046	[0.129, 0.310]		<0.001
Perceived benefits		0.117	0.043	[0.032, 0.202]		0.007
Perceived barriers		−0.097	0.034	[−0.164, −0.029]		0.005
Drug refusal self-efficacy		0.410	0.055	[0.302, 0.519]		<0.001
Self-control (BSCS)		0.108	0.044	[0.021, 0.196]		0.015

Abbreviations: B, unstandardized regression coefficient; SE, standard error; CI, confidence interval. Model 1 included sex, age, living arrangement, current smoking, risky drinking, subjective health status, and drug prevention education. Model 2 added the five Health Belief Model components: perceived susceptibility, perceived severity, perceived barriers, perceived benefits, and drug refusal self-efficacy. Model 3 added self-control. Reference categories were female sex, living with parents, not currently smoking, no risky drinking, good subjective health status, and having received drug prevention education. Independent living included living alone and in a dormitory residence. The final model was statistically significant, F(14, 265) = 26.69, *p* < 0.001, R^2^ = 0.585, adjusted R^2^ = 0.563. The Durbin–Watson statistic was 2.17, and the maximum variance inflation factor was 2.06.

**Table 4:** Exploratory analyses of indirect effects between Health Belief Model components and drug avoidance intention through self-control (*n* = 280).

Predictor	Effect	Coefficient	SE	95% CI
Susceptibility	Total effect	−0.345	0.041	[−0.426, −0.265]
	Direct effect	−0.293	0.042	[−0.376, −0.210]
	Indirect via self-control	−0.052	0.016	[−0.085, −0.023]
Severity	Total effect	0.479	0.048	[0.385, 0.573]
	Direct effect	0.438	0.047	[0.346, 0.529]
	Indirect via self-control	0.042	0.015	[0.015, 0.076]
Benefits	Total effect	0.332	0.053	[0.227, 0.436]
	Direct effect	0.270	0.053	[0.166, 0.373]
	Indirect via self-control	0.062	0.018	[0.030, 0.101]
Barriers	Total effect	−0.006	0.048	[−0.101, 0.088]
	Direct effect	0.008	0.045	[−0.081, 0.097]
	Indirect via self-control	−0.014	0.018	[−0.052, 0.018]
Self-efficacy	Total effect	0.630	0.043	[0.544, 0.715]
	Direct effect	0.585	0.044	[0.499, 0.672]
	Indirect via self-control	0.045	0.014	[0.018, 0.073]

Abbreviations: IDAS, Intention to Drug Avoidance Scale; BSCS, Brief Self-Control Scale; CI, confidence interval. Each Health Belief Model component was entered as the independent variable in a separate model, with self-control (BSCS) specified as the intervening variable and drug avoidance intention (IDAS) as the outcome. All models were adjusted for sex, age, living arrangement, current smoking, risky drinking, subjective health status, and drug prevention education. Standard errors and 95% confidence intervals for total and direct effects were obtained from the regression models. Indirect effects were estimated using 5000 bootstrap resamples, with bootstrap standard errors and percentile 95% CIs. An indirect effect was considered statistically significant when the 95% bootstrap CI did not include zero. These models did not adjust for the remaining Health Belief Model constructs; corresponding adjusted estimates are presented in Supplementary Table S2.

## Data Availability

The raw data supporting the conclusions of this article will be made available by the authors upon request due to privacy reasons.

## Supplementary Materials

The following supporting information can be downloaded at https://www.mdpi.com/article/10.3390/bs16091653/s1, Table S1: Differences in drug avoidance intention according to participant characteristics; Table S2: Indirect effects of Health Belief Model constructs on drug avoidance intention through self-control, adjusted for the remaining Health Belief Model constructs; Table S3: Completed Checklist for Reporting Results of Internet E-Surveys (CHERRIES).

## Abbreviations

The following abbreviations are used in this manuscript:

BSCS	Brief Self-Control Scale
CI	Confidence Interval
HBM	Health Belief Model
IDAS	Intention to Drug Avoidance Scale
