# Supplementary material for: Associations of Health Belief Model Constructs and Self-Control with Drug Avoidance Intention Among University Students in the Republic of Korea: A Cross-Sectional Study
Source: Behav Sci (Basel). 2026 Sep 15;16(9):1653. doi: 10.3390/bs16091653 (PMC13603294; doi:10.3390/bs16091653)
Supplement: Supplementary file 1 [file behavsci-16-01653-s001.zip › behavsci-4486916-supplementary.pdf]

Supplementary Table S1. Differences in drug avoidance intentions according to participant characteristics (N = 280)

Characteristic	Category	IDAS, Mean ± SD	t or F	*p*
Sex	Male	4.30 ± 0.55	2.35	.020
	Female	4.11 ± 0.68		
Living arrangement	With parents	4.22 ± 0.66	-0.12	.906
	Independent	4.23 ± 0.53		
Subjective health status	Poor [a]	4.26 ± 0.57	3.68	.026
	Fair [b]	4.14 ± 0.60		
	Good [c]	4.36 ± 0.62		
Perceived stress	Low	4.32 ± 0.58	1.44	.238
	Moderate	4.22 ± 0.64		
	High	4.17 ± 0.60		
Current smoking	Yes	3.97 ± 0.65	-2.52	.015
	No	4.26 ± 0.60		
Risky drinking	Yes	3.74 ± 0.67	-2.36	.041
	No	4.24 ± 0.60		
Drug prevention education	Yes	4.19 ± 0.64	-0.60	.547
	No	4.24 ± 0.60		

Abbreviation: IDAS, Intention to Drug Avoidance Scale. *p*-values for two-group comparisons were obtained using Welch's t-test, and one-way analysis of variance was used for subjective health and perceived stress. Independent living combines living alone and dormitory residence. Post hoc analysis indicated that the fair subjective health group had a significantly lower IDAS score than the good subjective health group (b < c, $p < .05$).

Supplementary Table S2. Indirect effects of Health Belief Model constructs on drug avoidance intention through self-control, adjusted for the remaining Health Belief Model constructs (N = 280)

Predictor (X)	a (X → SC)	b (SC → IDAS)	Direct effect (c′)	Indirect effect (a × b)	Boot SE	95% Boot CI
Perceived susceptibility	−0.212	0.108	−0.077	−0.023	0.013	[−0.055, −0.003]
Perceived severity	0.028	0.108	0.220	0.003	0.007	[−0.011, 0.018]
Perceived benefits	0.210	0.108	0.117	0.023	0.012	[0.003, 0.048]
Perceived barriers	−0.064	0.108	−0.097	−0.007	0.007	[−0.023, 0.003]
Drug refusal self-efficacy	0.041	0.108	0.410	0.005	0.009	[−0.014, 0.023]

Abbreviations: SC, self-control (Brief Self-Control Scale); IDAS, Intention to Drug Avoidance Scale; CI, confidence interval. Each Health Belief Model construct was entered as the independent variable in a separate model, with self-control specified as the intervening variable and drug avoidance intention as the outcome. In addition to sex, age, living arrangement, current smoking, risky drinking, subjective health status, and drug prevention education, the remaining four Health Belief Model constructs were

entered as covariates in both the self-control and outcome equations. Coefficients are unstandardized. Indirect effects were estimated using 5,000 bootstrap resamples with bootstrap standard errors and percentile 95% CIs, and were considered statistically significant when the CI did not include zero. Unadjusted estimates are presented in Table 4 of the main text.

Supplementary Table S3. Checklist for Reporting Results of Internet E-Surveys (CHERRIES)

CHERRIES category	Checklist item	Study-specific reporting
Design	Describe survey design	Cross-sectional, closed web-based survey of undergraduate students aged 19–26 years in the Republic of Korea, recruited from a commercial non-probability, opt-in online panel.
IRB approval and informed consent process	IRB approval	The original protocol and subsequent amendment were approved by the Institutional Review Board of Hallym University (HIRB-2025-136 and HIRB-2025-136-R, respectively).
	Informed consent	Electronic study information and informed consent were presented before questionnaire access. Participation was voluntary, and participants could discontinue the survey at any time.
	Data protection	The analytic dataset contained no personally identifiable information. Study data were stored on password-protected electronic devices.
Development and pre-testing	Development and testing	The research team pre-tested the web questionnaire before fielding to confirm technical functionality and survey flow.

Recruitment process and sample access	Open survey versus closed survey	Closed survey; access was limited to invited members of the Macromill Embrain research panel.
Recruitment process and sample access	Contact mode	Targeted electronic invitations were distributed by the panel provider to registered members aged 19–26 years. University-student status was verified at survey entry; respondents who did not select "university student" were screened out.
	Advertising the survey	No public advertising was used; recruitment was limited to targeted invitations to panel members.
Survey administration	Web/E-mail	The questionnaire was administered as a web-based survey through the commercial online research panel.
	Context	Access was restricted to invited Macromill Embrain panel members and the survey was not posted on a publicly accessible website.
	Mandatory/voluntary	Participation was voluntary. After consent, responses to all displayed items were required to proceed to the next screen, although participants could discontinue the survey at any time.
	Incentives	Panel reward points were provided to participants who completed the survey according to the provider's standard compensation policy.

	Time/Date	Initial data collection: April 21–27, 2026. After IRB approval of a protocol amendment, supplemental recruitment of 60 female participants was conducted July 16–17, 2026.
	Randomization of items or questionnaires	No item or questionnaire randomization was used; items were presented in a fixed sequence.
	Adaptive questioning	Eligibility screening used conditional termination for respondents who were not university students. Main study measures were presented in a fixed sequence.
	Number of items per screen	Displayed items varied by screen (approximately 1–7). Screening and demographic questions were generally presented individually; multi-item scales were presented in matrix format.
	Number of screens (pages)	The web questionnaire comprised 51 screens.
	Completeness check	All displayed items had to be answered before proceeding. Some sensitive items included a response-refusal or prefer-not-to-answer option.
	Review step	Participants could use the Back function to return to previous screens and modify responses before final submission.
Response rates	Unique site visitor	Unique participation was identified through the panel provider's unique panel identification system.

	View rate	Not calculated. This was a closed panel survey, and a public site-visitor denominator was not applicable.
	Participation rate	Not calculated. Invitations were targeted by registered age before undergraduate eligibility was verified at survey entry; the number of invited members meeting all eligibility criteria was unavailable.
	Completion rate	Not calculated because available provider records did not supply a denominator corresponding to participants who agreed to participate or started the questionnaire as defined by CHERRIES.
Preventing multiple entries from the same individual	Cookies used	Cookie-based duplicate prevention was not relied upon; duplicate participation was restricted through the unique panel identification system.
	IP check	No IP-address-based exclusion procedure was reported by the panel provider.
	Log file analysis	No additional log-file-based duplicate-entry procedure was reported by the panel provider.
	Registration	Unique panel IDs were used to prevent repeat participation; members who had already participated were prevented from re-entering the survey.

Analysis	Handling of incomplete questionnaires	Questionnaires discontinued before completion were not included in the analytic dataset. Only completed questionnaires meeting the study and data-quality criteria were analyzed.
	Data-quality screening	The panel provider excluded responses showing straight-lining across 30 or more consecutive Likert-type items or open-ended responses judged to be irrelevant, implausible, or otherwise indicative of inattentive responding.
	Questionnaires submitted with an atypical timestamp	No response-time cutoff was reported by the panel provider as an exclusion criterion.
	Statistical correction	No weighting, propensity-score adjustment, or other statistical correction for the non-probability panel sample was applied.
